# Complexities of Hemifacial Microsomia: A Case Study of Mandibular Hypoplasia and Ear Deformity

**DOI:** 10.7759/cureus.64499

**Published:** 2024-07-13

**Authors:** Shreya Khandelwal, Rajasbala Dhande, Pratapsingh Parihar, Anshul Sood

**Affiliations:** 1 Radiodiagnosis, Jawaharlal Nehru Medical College, Datta Meghe Institute of Higher Education and Research, Wardha, IND

**Keywords:** congenital disorder, multidisciplinary approach, craniofacial anomaly, ear deformity, mandibular hypoplasia, hemifacial microsomia

## Abstract

Hemifacial microsomia (HFM) presents a complex congenital anomaly characterized by the asymmetric underdevelopment of facial structures, predominantly affecting the ear, mouth, and mandible on one side of the face. This case study examines the intricacies of HFM through the presentation of a 23-year-old female with congenital deformities of the left ear and mandibular hypoplasia. Clinical and radiological evaluations revealed significant facial malformations, including mandibular hypoplasia, left temporomandibular joint fusion, and maxillary abnormalities, confirming the diagnosis of HFM. Management of HFM necessitates a multidisciplinary approach involving otolaryngologists, maxillofacial surgeons, orthodontists, and audiologists to comprehensively address functional and aesthetic concerns. Early diagnosis and intervention, along with psychosocial support, are essential for optimizing outcomes and improving the quality of life for individuals with HFM.

## Introduction

Hemifacial microsomia (HFM) is a congenital disorder characterized by the asymmetric underdevelopment of the facial structures, primarily affecting the ear, mouth, and mandible on one side of the face. It is the second most common craniofacial anomaly after cleft lip and palate, with an incidence rate ranging from one in 3,500 to one in 5,600 live births. The condition can present with varying degrees of severity, from mild facial asymmetry to significant deformities involving multiple craniofacial structures [1]. The etiology of HFM is believed to be multifactorial, involving genetic and environmental factors. It has been associated with disruptions in the development of the first and second pharyngeal arches during embryogenesis. These disruptions can result from vascular anomalies, teratogenic exposures, or genetic mutations, although the exact mechanisms remain unclear [2].

Clinically, HFM is characterized by a spectrum of anomalies. The most common features include mandibular hypoplasia, microtia or anotia (malformed or absent ear), and conductive hearing loss due to middle ear anomalies. Additional features may include soft tissue deficiencies, facial nerve involvement, and dental abnormalities [3]. The condition can be classified using the OMENS system, which stands for Orbital, Mandibular, Ear, Nerve, and Soft tissue involvement, providing a comprehensive framework for evaluating the extent of the deformity [4]. The diagnosis of HFM is primarily clinical, supplemented by imaging studies such as computed tomography (CT) or magnetic resonance imaging (MRI) to assess the extent of bony and soft tissue involvement. These imaging modalities are crucial for surgical planning and management [5]. The treatment of HFM requires a multidisciplinary approach, including surgical interventions to correct mandibular and maxillary deficiencies, reconstructive procedures for ear anomalies, and orthodontic treatments to address dental issues. Audiological management is also essential for addressing hearing impairments associated with the condition [6].

## Case presentation

A 23-year-old female presented with a congenital deformity of the left ear, which has been present since birth. She reported an absence of the left ear canal and reduced hearing on the same side, conditions she has lived with since birth. The patient was delivered via normal vaginal delivery and achieved all developmental milestones on time.

**Figure 1:**
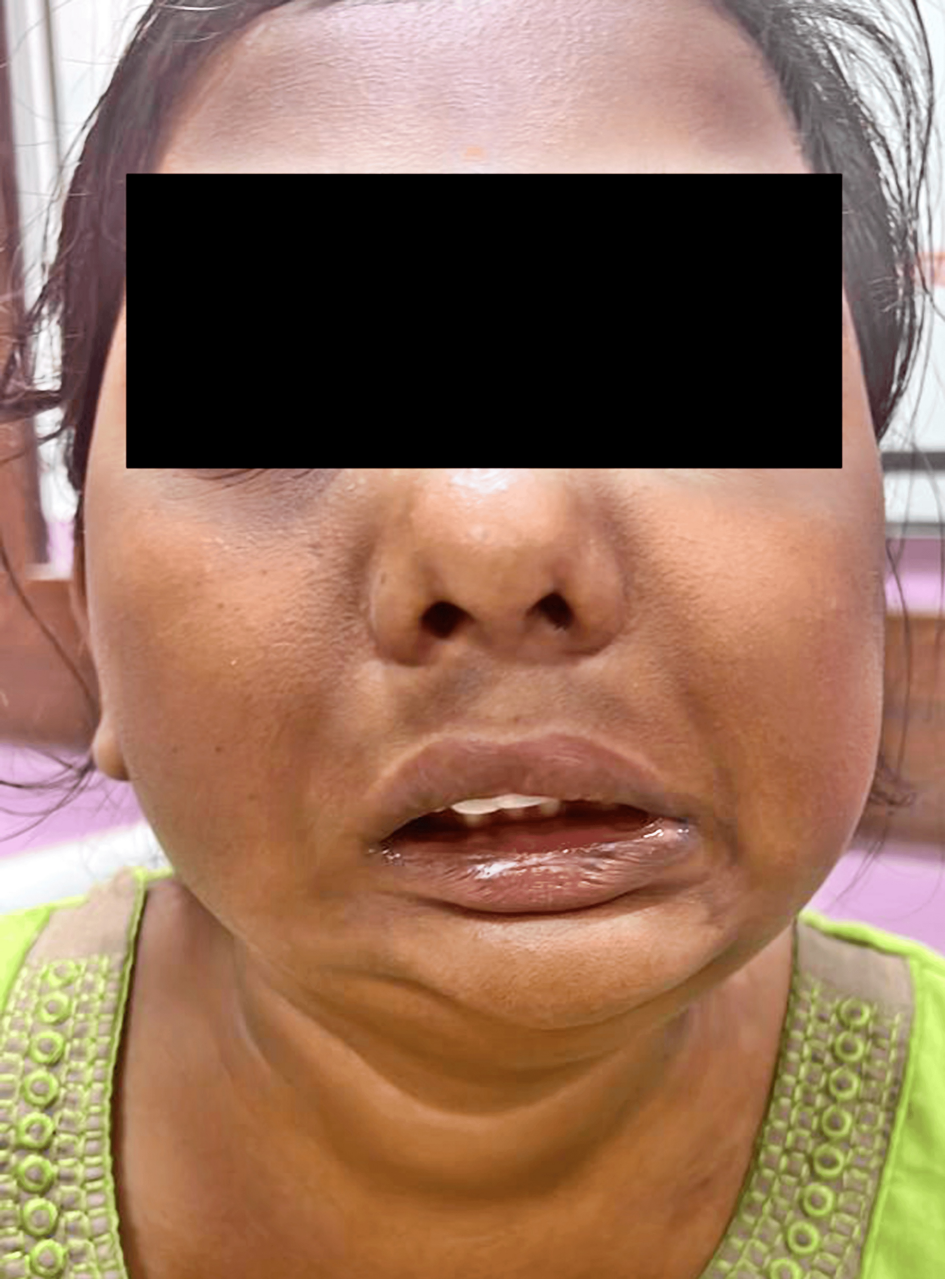
Facial malformation due to mandibular hypoplasia, resulting in a small, receding chin.

**Figure 2:**
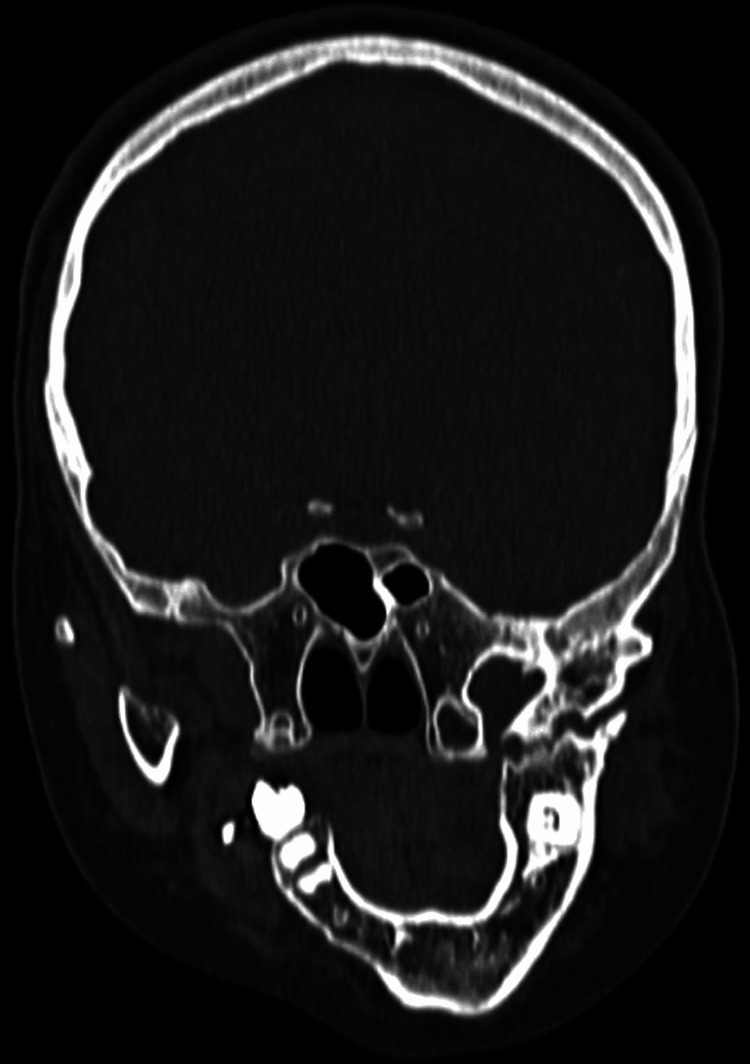
The right hemi-mandible appeared to be positioned medially compared to its anatomical position, indicating an asymmetry.

A CT brain plain scan was conducted upon radiological evaluation, revealing significant findings. The scan demonstrated facial malformation due to mandibular hypoplasia, resulting in a small, receding chin Figure 1. The left temporomandibular joint was fused with the left coronoid process extending into the anterior temporal fossa. A breach in the continuity of the ramus of the left hemi-mandible with sclerosed margins, suggestive of pseudoarthrosis, was noted. The right hemi-mandible was positioned medially compared to its anatomical position, indicating an asymmetry Figure 2. Further examination revealed that the left zygomatic arch was shortened, and the maxilla was reduced in height. There was also mucosal thickening in the left maxillary sinus, with the root of a molar tooth noted in the sinus, suggesting odontogenic sinusitis.

These findings indicate HFM, a congenital disorder characterized by the underdevelopment of one side of the face. In this case, the patient's presentation included deformity of the left ear, absence of the left ear canal, mandibular hypoplasia, and associated facial asymmetry. HFM can have varying severity and affect the ear, mouth, and jaw, leading to functional and aesthetic issues.

Managing this patient's condition necessitates a multidisciplinary approach involving specialists such as otolaryngologists, maxillofacial surgeons, orthodontists, audiologists, and speech therapists. Key considerations include a comprehensive audiometric assessment to determine the degree of hearing loss and explore hearing aid options, including bone-anchored hearing aids (BAHA) or cochlear implants. Surgical interventions might be required to address the mandibular hypoplasia, temporomandibular joint abnormalities, and potential corrections to the zygomatic arch and maxilla to improve facial symmetry. Dental and orthodontic care is also crucial, particularly in managing odontogenic sinusitis and other related dental issues.

## Discussion

HFM presents a multifaceted challenge in both diagnosis and management due to its variable presentation and involvement of multiple craniofacial structures. This discussion aims to explore the complexities of HFM, emphasizing the significance of a multidisciplinary approach in providing comprehensive care to affected individuals. The diagnosis of HFM often relies on clinical evaluation supplemented by imaging studies such as CT or MRI [7]. These modalities enable clinicians to assess the extent of bony and soft tissue involvement, facilitating accurate diagnosis and surgical planning. In the case presented, CT imaging revealed mandibular hypoplasia, temporomandibular joint abnormalities, and maxillary deformities, confirming the diagnosis of HFM. A fundamental aspect of managing HFM is understanding its etiology, which is believed to be multifactorial, involving genetic and environmental factors [8]. Disruptions in the development of the first and second pharyngeal arches during embryogenesis play a crucial role in the pathogenesis of HFM [9]. While the exact mechanisms remain unclear, vascular anomalies, teratogenic exposures, and genetic mutations have been implicated in the development of this condition.

The management of HFM necessitates a multidisciplinary approach, encompassing various specialties such as otolaryngology, maxillofacial surgery, orthodontics, and audiology [10]. Each aspect of care requires tailored interventions to address functional and aesthetic concerns comprehensively. Surgical interventions may include mandibular reconstruction, ear reconstruction, and correction of maxillary and zygomatic deformities to improve facial symmetry and function [11]. Audiological management is essential in individuals with HFM due to the high prevalence of conductive hearing loss associated with middle ear anomalies [12]. Comprehensive audiometric assessments help determine the degree of hearing loss and guide the selection of appropriate interventions, such as BAHA or cochlear implants, to optimize auditory function. Orthodontic care plays a crucial role in managing dental anomalies associated with HFM, including malocclusion and dental crowding [13]. Timely orthodontic interventions help address these issues and contribute to the overall facial aesthetics and function of affected individuals. Psychosocial support is integral to the care of individuals with HFM, given the potential impact of facial deformities on self-esteem and quality of life [14]. Counseling and support groups provide valuable resources for patients and their families, facilitating coping mechanisms and enhancing psychosocial well-being.

## Conclusions

In conclusion, HFM presents a complex clinical scenario requiring a multidisciplinary approach for optimal management. The case study illustrates the diagnostic challenges and treatment complexities inherent in this congenital disorder, characterized by asymmetric facial underdevelopment. Through collaborative efforts among otolaryngologists, maxillofacial surgeons, orthodontists, and audiologists, tailored interventions address functional deficits, aesthetic concerns, and psychosocial implications. Early diagnosis, supplemented by imaging studies, facilitates the timely initiation of surgical, audiological, and orthodontic interventions aimed at improving facial symmetry, restoring auditory function, and enhancing overall quality of life. Psychosocial support plays a pivotal role in supporting patients and their families, fostering coping mechanisms, and promoting resilience in the face of the challenges posed by HFM. By integrating expertise from diverse specialties and providing personalized care, clinicians can optimize outcomes and strive toward improving the holistic well-being of individuals affected by HFM.
